# Correction: Si et al. Research on the Influence of Non-Cognitive Ability and Social Support Perception on College Students’ Entrepreneurial Intention. *Int. J. Environ. Res. Public Health* 2022, *19*, 11981

**DOI:** 10.3390/ijerph22060959

**Published:** 2025-06-19

**Authors:** Wentao Si, Jiayi Tian, Qi Yan, Wenshu Wang, Maocong Zhang

**Affiliations:** 1 School of Public Administration, Shandong Normal University, Jinan 250014, China201829010205@stu.sdnu.edu.cn (J.T.);; 2 Research Center for Educational Policy and Management, Shandong Normal University, Jinan 250014, China

## Addition and Removal of Authors

In the original publication [1], **Jiayi Tian** was not included as an author in the original publication. **Lin Meng** was included as an author in the original publication. The corrected Author Contributions statement appears here. The authors state that the scientific conclusions are unaffected. This correction was approved by the Academic Editor. The original publication has also been updated.
